# Comprehensive SPECT/CT system characterization and calibration for ^177^Lu quantitative SPECT (QSPECT) with dead-time correction

**DOI:** 10.1186/s40658-020-0275-6

**Published:** 2020-02-14

**Authors:** Andrea Frezza, Corentin Desport, Carlos Uribe, Wei Zhao, Anna Celler, Philippe Després, Jean-Mathieu Beauregard

**Affiliations:** 1 Cancer Research Center, Université Laval, Quebec City, QC Canada; 2 Department of Physics, Engineering Physics and Optics, Université Laval, Quebec City, QC Canada; 3 Oncology Division, CHU de Québec – Université Laval Research Center, Quebec City, QC Canada; 4 Functional Imaging Department, BC Cancer, Vancouver, BC Canada; 5 Medical Imaging Research Group, Department of Radiology, University of British Columbia, Vancouver, BC Canada; 6 Department of Radiation Oncology, CHU de Québec – Université Laval, Quebec City, QC Canada; 7 Department of Radiology and Nuclear Medicine, Université Laval, Quebec City, QC Canada; 8 Department of Medical Imaging, CHU de Québec – Université Laval, 11 côte du Palais, Quebec City, QC G1R 2J6 Canada

**Keywords:** Quantitative SPECT, SPECT/CT, ^177^Lu, Calibration, Dead time

## Abstract

**Background:**

Personalization of ^177^Lu-based radionuclide therapy requires implementation of dosimetry methods that are both accurate and practical enough for routine clinical use. Quantitative single-photon emission computed tomography/computed tomography (QSPECT/CT) is the preferred scanning modality to achieve this and necessitates characterizing the response of the camera, and calibrating it, over the full range of therapeutic activities and system capacity. Various methods to determine the camera calibration factor (*CF*) and the deadtime constant (τ) were investigated, with the aim to design a simple and robust protocol for quantitative ^177^Lu imaging.

**Methods:**

The SPECT/CT camera was equipped with a medium energy collimator. Multiple phantoms were used to reproduce various attenuation conditions: rod sources in air or water-equivalent media, as well as a Jaszczak phantom with inserts. Planar and tomographic images of a wide range of activities were acquired, with multiple energy windows for scatter correction (double or triple energy window technique) as well as count rate monitoring over a large spectrum of energy. Dead time was modelled using the paralysable model. *CF* and τ were deduced by curve fitting either separately in two steps (*CF* determined first using a subset of low-activity acquisitions, then τ determined using the full range of activity) or at once (both *CF* and τ determined using the full range of activity). Total or segmented activity in the SPECT field of view was computed. Finally, these methods were compared in terms of accuracy to recover the known activity, in particular when planar-derived parameters were applied to the SPECT data.

**Results:**

The SPECT camera was shown to operate as expected on a finite count rate range (up to ~ 350 kcps over the entire energy spectrum). *CF* and τ from planar (sources in air) and SPECT segmented Jaszczak data yielded a very good agreement (*CF* < 1% and τ < 3%). Determining *CF* and τ from a single curve fit made dead-time-corrected images less prone to overestimating recovered activity. Using triple-energy window scatter correction while acquiring one or more additional energy window(s) to enable wide-spectrum count rate monitoring (i.e. ranging 55–250 or 18–680 keV) yielded the most consistent results across the various geometries. The final, planar-derived calibration parameters for our system were a *CF* of 9.36 ± 0.01 cps/MBq and a τ of 0.550 ± 0.003 μs. Using the latter, the activity in a Jaszczak phantom could be quantified by QSPECT with an accuracy of 0.02 ± 1.10%.

**Conclusions:**

Serial planar acquisitions of sources in air using an activity range covering the full operational capacity of the SPECT/CT system, with multiple energy windows for wide-spectrum count rate monitoring, and followed by simultaneous determination of *CF* and τ using a single equation derived from the paralysable model, constitutes a practical method to enable accurate dead-time-corrected QSPECT imaging in a post-^177^Lu radionuclide therapy setting.

## Background

^177^Lu-DOTA-octreotate peptide receptor radionuclide therapy (PRRT) is an effective palliative treatment for neuroendocrine tumours [1, 2]. So far, PRRT has mostly been practised in an empiric fashion (e.g. four cycles of 7.4 GBq ^177^Lu-DOTA-octreotate), despite the well-known high inter-patient variability of absorbed doses to healthy tissues per injected activity [3–5]. There is growing evidence that personalizing PRRT based on image-based dosimetry calculations could enhance its efficacy without augmenting toxicity, by increasing injected activity and tumour irradiation in a majority of patients, while limiting radiation exposure of their healthy tissues [3–5]. Dosimetry-based personalization could also benefit the rapidly developing prostate-specific membrane antigen (PSMA) radioligand therapy (RLT) with ^177^Lu [6]. Quantitative single-photon emission computed tomography/computed tomography [(Q)SPECT/CT] overcomes many limitations of planar imaging and is emerging as the preferred scanning method to perform internal dosimetry of ^177^Lu-based radionuclide therapy [7–9].

Accurate quantification is possible if corrections for image degrading effects are applied and the camera system is characterized and calibrated over the entire range of activities used in clinical practice [10]. Compensation for scatter and attenuation, which are widely available on current SPECT/CT systems, are essential for QSPECT [9–15]. In addition, dead-time (DT) correction is needed to maximize the accuracy of ^177^Lu quantification and this correction requires determination of the camera DT constant (τ) [8]. Finally, in order to convert the SPECT image counts into activity concentration, the camera calibration factor (*CF*) must be measured. Determination of both, *CF* and τ, requires experiments with ^177^Lu sources and/or phantoms. This characterization must be performed for each combination of radionuclide, collimator, energy window setting and camera [9].

Several methods have been proposed to evaluate these parameters, some more demanding than others in terms of acquisition time, decay period, and image processing [8, 9, 16–21]. Planar imaging-based calibration is faster and more convenient to execute and is expected to yield accurate *CF* [9, 20] relative to fully tomographic calibration as the reference method [9, 21, 22]. The objectives of this study were to perform a comprehensive characterization of our SPECT/CT system’s response (combined effects of *CF* and τ) over its full range of quantifiable ^177^Lu activities and to compare various acquisition and analysis methods for camera calibration for QSPECT imaging, with the aim to simplify this process. In particular, we investigated: (1) how accurate *CF* and τ determined in planar mode are when applied to reconstructed SPECT images of phantoms of varied geometry; (2) if *CF* is more conveniently determined separately at low activity, or simultaneously to τ over the full range of quantifiable activity; and (3) if DT losses of primary photon counts—i.e. scatter-corrected photopeak counts, whether from planar or reconstructed SPECT images—could be practically estimated from wide-energy spectrum acquisition counting rate, as presented in [8], as opposed to having to determine DT for each of the three windows used for triple energy window (TEW) scatter correction as presented in [21], or for just the photopeak window as presented by Willowson et al. [23]. Also, we evaluated if segmenting the SPECT images to remove spurious counts in non-radioactive and dense areas of phantoms improves quantitative accuracy with which total activity of the phantom can be recovered, as well as the impact of using triple vs. double energy window (DEW) scatter correction, and that of reducing the width of the recorded wide-energy spectrum window for DT determination.

## Materials and methods

### SPECT/CT system

A dual-head Symbia T6 SPECT/CT system (Siemens Healthineers, Erlangen, Germany) with a NaI crystal thickness of 9.5 mm and equipped with a medium energy low penetration collimator was used.

### Energy windows

**Fig. 1:**
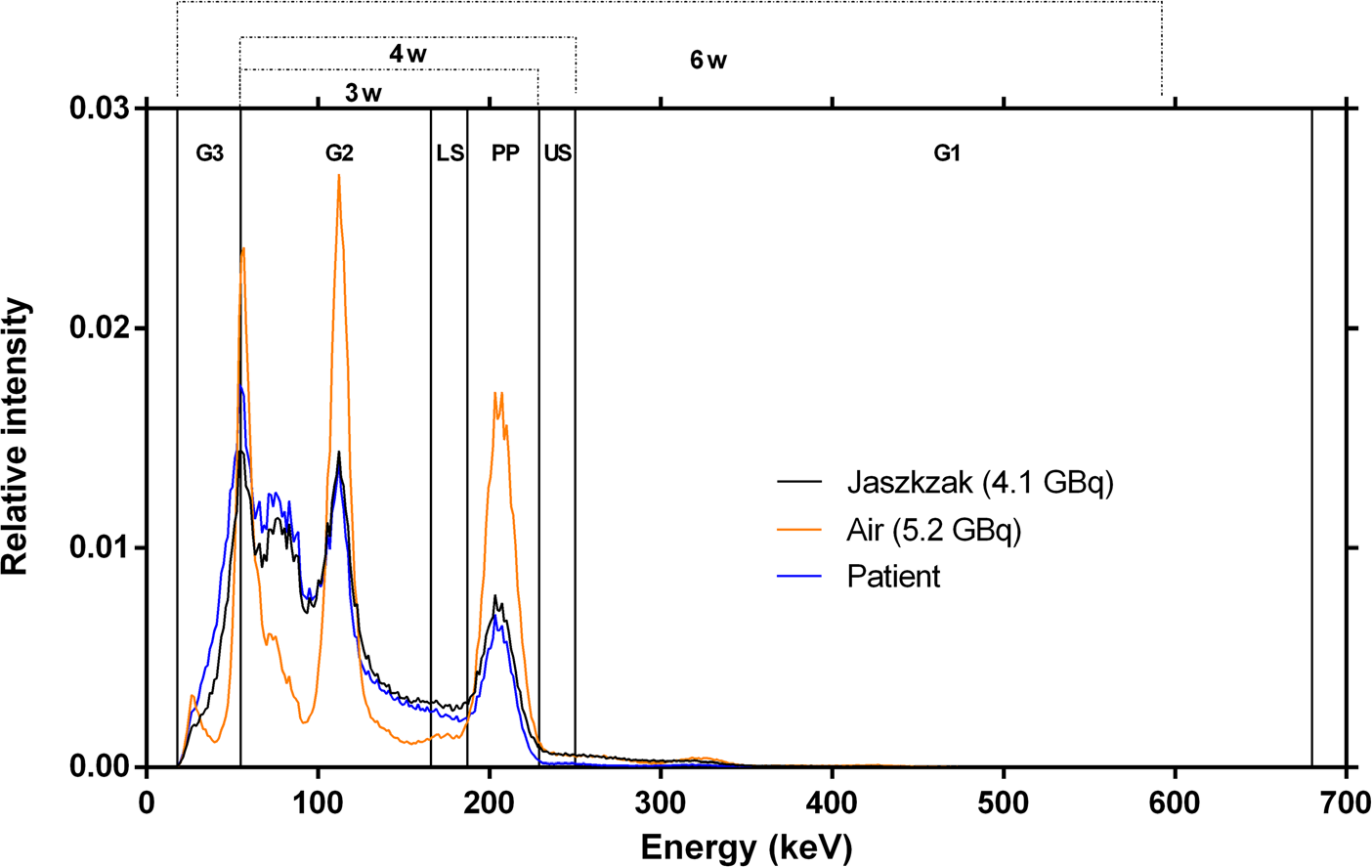
^177^Lu energy spectrum (normalized to the area under the curve) for sources in air (5.2 GBq), the Jaszczak phantom (4.1 GBq), and a patient (treated with 4.7 GBq ^177^Lu-octreotate). The energy windows and combinations thereof, described in Table 1, are also shown

The energy window settings, allowing us to perform double (DEW) or triple (TEW) energy window scatter correction [11], are detailed in Table 1. Either the photopeak window only, or a combination of 3, 4 or 6 contiguous energy windows (*3W*, *4W* and *6W*, respectively) were used to monitor the observed acquisition counting rate (*R*_*Wo*_) and to assess whether DT could be accurately estimated using a narrower portion of the energy spectrum than *6W* (Fig. 1).

**Table 1:** Energy window settings and combinations used for acquisition and analysis

	Centre (keV)	Width	Limits [lower-upper] (keV)
Energy window settings			
PP (Photopeak)	208	20%	[187–229]
LS (Lower Scatter)		10%	[166–187]
US (Upper Scatter)		10%	[229–250]
G1 (General Scatter 1)	465	93%	[250–680]
G2 (General Scatter 2)	111	100%	[55–166]
G3 (General Scatter 3)	37	100%	[18–55]
Energy window combinations			
3W (PP + LS + G2)			[55–229]
4W (PP + LS + US+G2)			[55–250]
6W (PP + LS + US + G1 + G2 + G3)			[18–680]

**Fig. 2:**
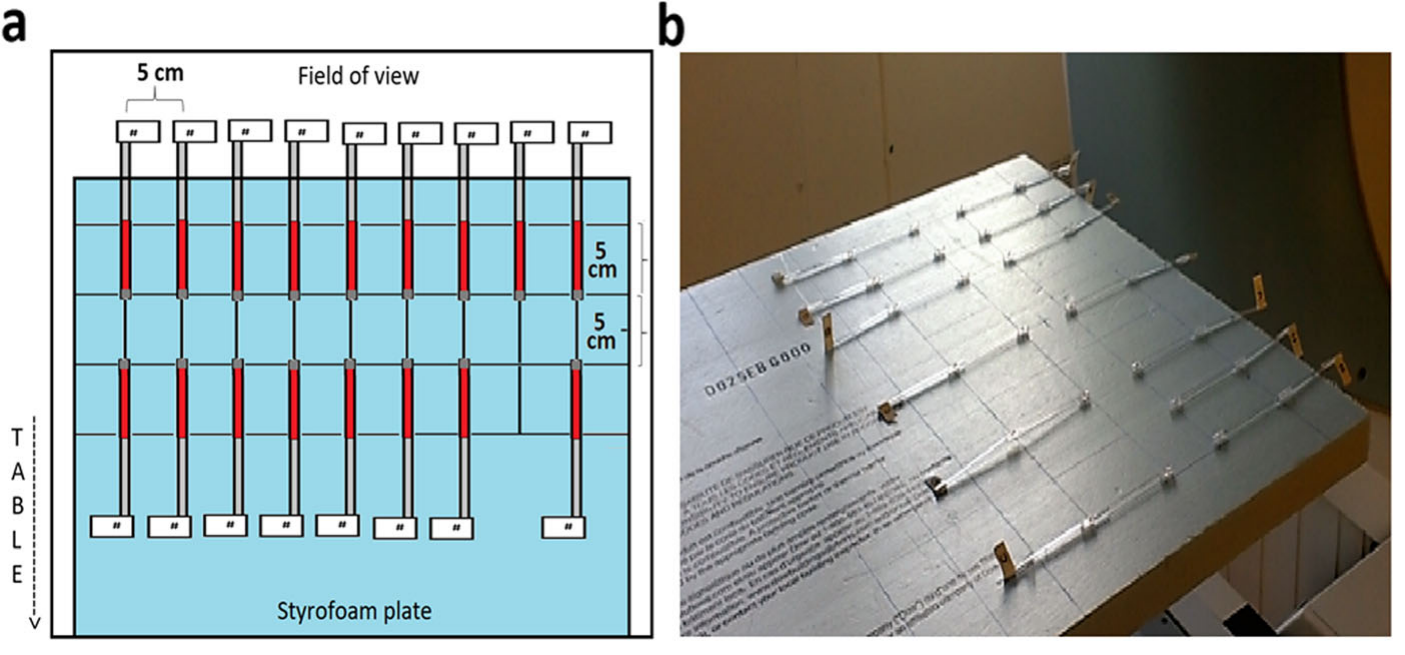
Schema (**a**) and picture (**b**) of capillary ^177^Lu sources disposition on the polystyrene foam board (Air-2D phantom) for planar acquisitions

Because photons of any energy, not only those recorded in the photopeak window, can cause camera DT [8, 21, 24], and because the shape of the energy spectrum changes depending on the geometry of the scanned object (in particular the volume of attenuating/scattering matter, Fig. 2), we acquired data in 6 contiguous energy windows covering practically all the energy range of ^177^Lu events recordable by our SPECT system (Table 1). By default, and unless otherwise specified, we used the summed count rate from these 6 energy windows (*6W*, 18–680 keV, Table 1) for wide-spectrum-based modelling of DT affecting primary counts. However, we hypothesize that acquiring only the *general scatter* windows that accumulates the largest fraction of counts (*G2*, 55-166 keV; Table 1) in addition to the photopeak and scatter windows would suffice to accurately monitor and correct for DT when scanning objects of varied geometries.

### Phantoms

To compare calibration and DT parameters obtained with planar vs. tomographic acquisitions, sources placed in air and extended phantoms were scanned. Only point sources in air were used with planar imaging (without attenuation correction). To emulate various attenuation and scatter conditions typically encountered in clinical SPECT studies, multiple phantoms were scanned using tomographic acquisitions.

Two types of containers were used to hold the activity: plastic capillary tubes (inner diameter: 1.1 mm, length: 7.5 cm) and a Jaszczak SPECT Deluxe phantom with cold rod inserts and fillable spheres (Data Spectrum Corporation, Durham, USA).

To characterize the DT response of the camera, a large range of ^177^Lu activities (^177^LuCl_3_ from IBD-Holland, The Netherlands) were used. The dose calibrator (Atomlab 400, Biodex, USA) was calibrated with a NIST traceable ^177^Lu source (1998 MBq, Eckert & Ziegler, Valencia, CA, USA) and used to measure the activity in the capillaries, and in the syringe before and after the filling of the Jaszczak and its spheres.

**Fig. 3:**
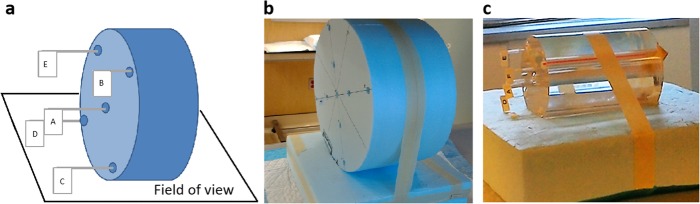
Schema (**a**) and pictures (**b**, **c**) of cylindrical phantoms used in SPECT acquisitions with capillary sources. The Air-3D phantom is shown in **b** while the CTDI-10 phantom is shown in **c**. Starting from the centre, the capillaries were added successively (up to three capillaries per hole)

Seventeen capillaries were filled over approximately 5 cm in length with concentrated ^177^LuCl_3_ solution (up to 34 GBq/mL) and sealed with wax. To each tube, a labelled stem was attached for easier source manipulation and identification. The choice of capillary tubes was motivated by the need to have small volumes filled with high activity spread over several pixels in order to avoid saturation (i.e. > 65,535 counts in one pixel or reconstructed voxel) [8]. The ranges of activities used in each phantom are presented in Table 2. The following phantom configurations were used:

1. Sources in air—2D layout (Air-2D): Capillary tubes were placed on a 2.5 × 50 × 70 cm^3^ polystyrene foam board (minimal attenuation, thus considered “air equivalent”) that was fixed to the end of the camera’s couch so that the capillaries were positioned at a distance of 34.6 cm from each camera head (Fig. 2).
2. Sources in air—3D layout (Air-3D): A cylindrical piece of polystyrene foam (32 cm in diameter) and with nine holes corresponding to the patterns of the adult head/pediatric body (CTDI-16) and adult body (CTDI-32) phantoms for measurement of CT dose indexes was manufactured (Fig. 3b). The capillary tubes were placed in these holes.
3. Sources in water equivalent medium (CTDI): Three polymethylmethacrylate cylindrical phantoms (diameters of 10, 16 and 32 cm, respectively), typically used for measurements of the CT dose index (CTDI-10, CTDI-16 and CTDI-32, respectively; Pycko Scientific Limited, Grantham, England [25];) were employed in these experiments. Each of these phantoms has five holes into which capillary tubes were inserted (Fig. 3c).
4. Sources in water with background activity (Jaszczak): The Jaszczak phantom was filled with a total of 19,111 MBq of ^177^Lu. Part of this activity (18,535 MBq) was diluted in 6.1 L of water and filled the main compartment of the phantom, with an excess of a chelating agent (diethylenetriamine pentaacetate, DTPA) to avoid precipitation of activity on the phantom walls [19]. The remaining 574 MBq were diluted in 31.8 mL of water and used to fill the spheres, resulting in a concentration ratio of 6:1 between the spheres and the cylinder (18 and 3 MBq/mL, respectively) [20].

**Table 2:** Ranges of activity and count rates, per phantom

Acquisition type	Phantom	Total no. of acquisitions^a^	Activity range (MBq)	Photopeak count rate range (cps)	Wide-spectrum (18–680 keV) count rate range (cps)
Planar	Air-2D	34	19–15,123	208–97,638	851–375,605
Tomographic	Air-3D	35	32–15,274	327–92,275	1342–370,033
	CTDI-10	21	13–12,053	93–64,027	665–367,557
	CTDI-16	17	16–13,540	83–61,456	695–377,300
	CTDI-32	21	21–18,625	50–60,328	513–380,170
	Jaszczak	30	44–18,985	307–55,270	2650–386,006

^a^This is the total number of acquisitions performed for each phantom, but only subsets of acquisitions with an activity within the usable range of the system were used to derive the calibration factor and dead-time constant (Tables 3 and 4)

### Acquisitions and reconstruction

A dynamic planar acquisition of the Air-2D phantom was performed while adding 17 capillary tubes to the board, one at a time, during every odd-numbered frame, so that the activity was stable during the even-numbered frames. This experiment was performed twice, 15 days apart, with frame durations of 30 and 60 s, respectively, yielding a series of 34 planar acquisitions. A matrix of 256 × 256 was used. The counts acquired in the 208 keV photopeak window were scatter-corrected using TEW method (or DEW, when specified), i.e. by subtracting the summed counts collected in the scatter window(s) from those recorded in the photopeak window, and then dividing the result by the frame duration, to obtain the observed *primary* photons count rate (*R*_*Po*_). The analyses were performed for each camera detector separately and also using averaged counts from both detectors.

Tomographic acquisitions were performed with the five 3D phantoms (Air-3D, CTDI-10, CTDI-16, CTDI-32, Jaszczak), using the same settings as used in our current clinical protocol: 96 projections (48 per head), step-and-shoot mode, non-circular orbit, 128 × 128 matrix (4.8-mm voxel). To avoid voxel saturation, the time per projection was adjusted according to the activity in the phantom and ranged from 1 to 24 s for Air-3D and CTDI phantoms, and from 1 to 180 s for the Jaszczak phantom. For Air-3D and CTDI phantoms, acquisitions were obtained with one to 15 capillaries placed in holes (up to 3 per hole), and were performed twice, 15 days apart. The Jaszczak phantom was scanned 30 times over 59 days.

All tomographic datasets were reconstructed using the vendor’s ordered subset expectation maximization iterative algorithm with resolution recovery (Flash3D, Siemens Healthineers, Erlangen, Germany), with 4 iterations and 8 subsets, a CT-based attenuation correction (110 kVp, 70 mAs CARE Dose 4D, B08s convolution kernel, extended field of view, coregistered to the SPECT volume and converted to a 208-keV attenuation map, narrow-beam geometry), and TEW (or DEW, when specified) scatter correction. Because the reconstructed images are scaled by the number of projections, *R*_*Po*_ was obtained by dividing the sum of counts in the reconstructed SPECT volume by the product of the number of projections and their duration.

Unless otherwise specified, primary photon counts (and thus *R*_*Po*_) were obtained by applying TEW scatter correction to photopeak counts recorded in both planar and tomographic mode. We previously used DEW scatter correction because of the lack of down-scatter from high-energy events [8], but more recently observed that pile-up events could accumulate in the upper scatter window at high activity [21]. We wanted to assess the impact of DEW vs. TEW on quantitative accuracy. We therefore re-analysed the data with simplified energy window schemes: 4 energy windows (*4 W*) and TEW; 3 energy windows (*3 W*) and DEW (Table 1).

### Segmentation

Initially*, R*_*Po*_ of the reconstructed SPECT images was computed using the entire volume of the phantom. However, particularly in the presence of a large volume of non-radioactive attenuating material, such as when using the CTDI phantoms, excess scattered counts may be ineffectively eliminated with DEW or TEW scatter correction techniques [20, 26]. In an attempt to compensate for this, the following segmentation techniques were applied to compute *R*_*Po*_:

- ROI method: For the Jaszczak phantom (which has an inner diameter of 21 cm), a 23-cm circular region of interest (ROI) was drawn on each slice of the phantom. For Air-3D and CTDI phantoms, up to five 3.8-cm circular ROIs were drawn on each slice containing the capillaries and centred on these.
- Threshold method: For all tomographic phantoms, a threshold segmentation approach was used, in which one percent of the maximum voxel value in the volume was used as the volume of interest lower threshold [17].

### Camera calibration factor and dead-time constant

The term *camera sensitivity* is defined as *R*_*Po*_ per known activity (*A*). Ideally, as the activity increases, the detected count rate should increase proportionately, and the sensitivity should remain constant. However, in scans where high activities are used, the sensitivity decreases because the count rate is affected by DT. We reserve the term *camera calibration factor* (*CF*) only to the DT-free data, i.e. when *R*_*Po*_ equals *R*_*Pt*_ (the true primary count rate), such as data obtained in very low counting rate conditions or after DT correction (Eq. 1), while the *camera sensitivity* combines *CF* and the DT effects. *CF* thus characterizes the system and is used to convert the reconstructed SPECT primary count rate data into activity after DT correction, and ultimately into activity concentration.

1 $$ CF={R}_{Pt}/A $$

DT response of modern gamma cameras is typically described by the paralysable model (Eq. 2 [21, 27, 28];).

2 $$ {R}_o={R}_t\cdot {e}^{-{R}_t\cdot \tau } $$

where *R*_*o*_ and *R*_*t*_ are the observed and true count rates, respectively.

We modified Eq. 2 to determine the DT affecting *R*_*Po*_ based on the observed acquisition count rate *R*_*Wo*_ in a given energy window *W*, (Eq. 3, as in reference [8], which can be re-written as Eq. 4).

3 $$ {R}_{Wo}= CF\cdot A\cdot \frac{R_{Wo}}{R_{Po}}\cdot {e}^{- CF\cdot A\cdot \frac{R_{Wo}}{R_{Po}}\cdot \tau } $$

4 $$ {R}_{Wo}=\frac{R_{Pt}}{R_{Po}}\cdot {R}_{Wo}\cdot {e}^{-\frac{R_{Pt}}{R_{Po}}\cdot {R}_{Wo}\cdot \tau } $$

In SPECT, *R*_*Wo*_ is computed by summing the counts within energy window *W* from all projections acquired by both detectors, divided by number of images and time per projection. It is important to note that Eqs. 3 and 4 do not imply that DT affecting *R*_*Po*_ is equal to that affecting and *R*_*Wo*_, and that the τ obtained here is only used to estimate the DT correction factor applying to *R*_*Po*_ (i.e. *R*_*Pt*_/*R*_*Po*_, Eq. 4) as a function of the acquisition counting rate *R*_*Wo*_, but not to correct *R*_*Wo*_ itself for DT. Because other phenomena such as pulse pile-up will distort the energy spectra histogram at higher count rates, primary photons count losses will be greater than the acquisitions count losses in energy window *W*, when *W* is significantly wider than the photopeak window, as some of the primary photons will be detected only outside of the photopeak window, resulting in some lost primary events still counted in *W*. Accordingly, it is expected that the *R*_*Wo*_-based τ to estimate DT affecting *R*_*Po*_ will be larger than a τ describing DT affecting *R*_*Wo*_ itself.

For planar and tomographic data, sensitivity was first plotted as a function of activity, photopeak count rate and wide-spectrum (*6W*) count rate. This allowed us to determine which parameter from these three best describes the DT behaviour of the SPECT camera independently of the attenuation and scatter conditions. For planar and tomographic data, we then determined *CF* and τ using two methods, A and B, with GraphPad Prism (v. 7.0, GraphPad Software, La Jolla, CA, USA).

- Method A (low-activity *CF*): *CF* and τ are determined in two steps. *CF* is first determined by plotting the observed primary photons count rate as a function of activity and performing a linear fit forced to cross the origin for DT-free data points (i.e. obtained at low count rate where less than 1% DT is observed). The slope of the fit represents *CF.* The next step was to fix *CF* in Eq. 3 and plot *R*_*Wo*_ against *X*_*W*_, where ${X}_W=A\bullet \frac{R_{Wo}}{R_{Po}}$. Data was fit (non-linear curve fit) to Eq. 3 with fixed *CF* to determine τ.
- Method B (full-range *CF*): Both the *CF* and τ are determined simultaneously, by plotting *R*_*Wo*_ versus *X*_*W*_ and fitting the data (non-linear curve fit) to Eq. 3. An advantage of Method B over Method A is that camera *CF* is determined by using the full range of activity, without the need for an *a priori* assumption that any dataset is DT-free. Indeed, the activity or count rate thresholds below which DT is negligible is usually unknown beforehand.

### Accuracy

For each phantom, with each acquisition, scatter correction and segmentation methods applied, the quantitative accuracy (i.e. percentage deviation of recovered activity from images from the known activity) was first evaluated when using the *CF* and τ derived from the respective set of conditions. Secondly, quantitative accuracy of SPECT data was assessed using *CF* and τ derived from planar calibration, in order to validate the latter as a practical calibration method.

## Results

### Planar-derived calibration factor and dead-time constant

**Fig. 4:**
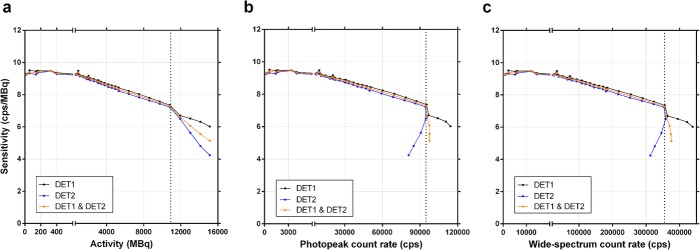
Planar sensitivity (observed primary photons count rate per activity) as a function of activity (**a**), of photopeak count rate (187–229 keV; **b**) or 6-window wide-spectrum count rate (18–680 keV; **c**). The dotted line indicates the upper limit of the usable range. Data is shown for each detector separately, and averaged

The planar sensitivity was plotted as a function of activity and of photopeak and wide spectrum count rates (Fig. 4). Even at low activity, the sensitivity is not affected by the background counts as the latter are efficiently eliminated along with the scatter counts by the scatter correction in planar mode (i.e. total image scatter counts subtracted from total image photopeak counts). Above a certain activity level, and the corresponding count rate levels (dotted lines), both detectors exhibited a sharp drop in sensitivity, and then a divergent behaviour, with Detector 2 suffering a more pronounced decrease in sensitivity. According to our SPECT/CT system vendor, this is due to the design of the system which results in Detector 1 being prioritized at high count rate. The dotted lines thus represent the maximum unattenuated activity which can be reliably quantified (10.8 GBq), and the maximum usable photopeak (94 kcps) and wide-spectrum (355 kcps) count rates of the system.

**Fig. 5:**
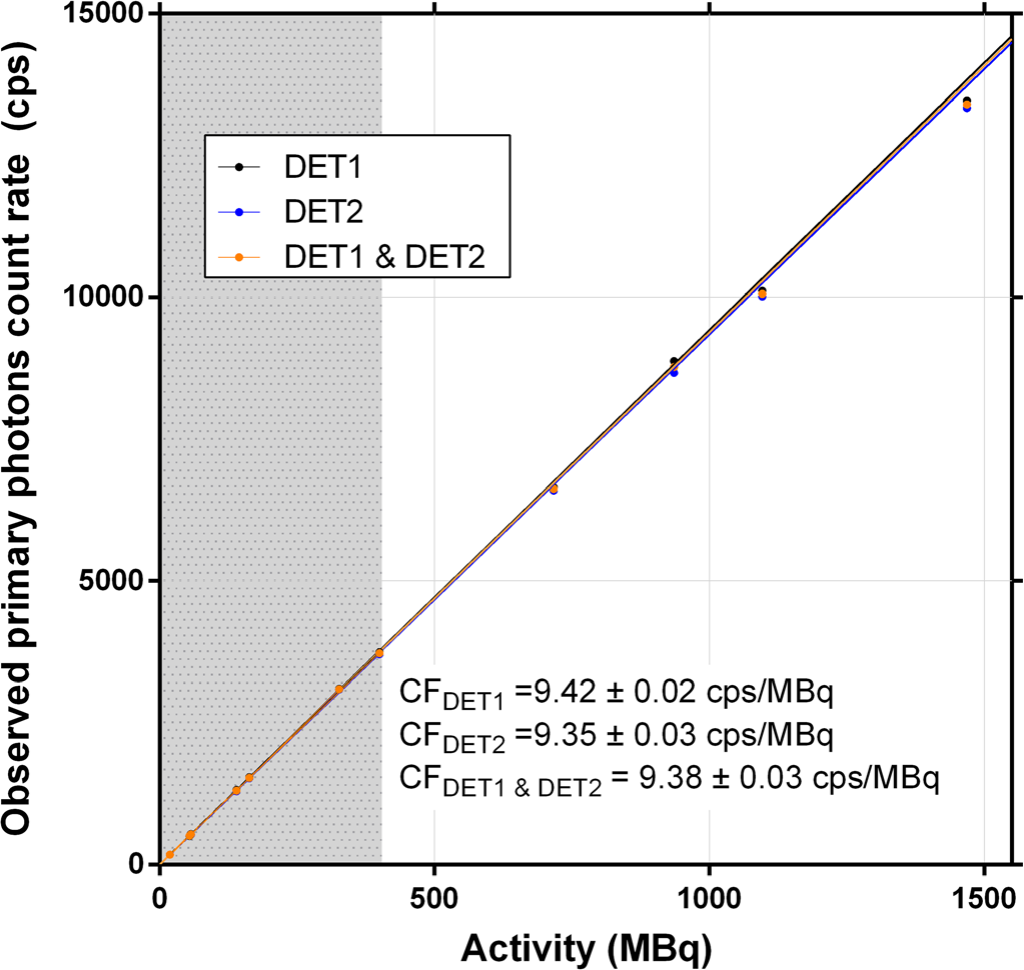
Observed primary photons count rate vs. activity for planar acquisitions. The calibration factor (*CF*) was obtained by linear fit forced through origin of low-activity data, i.e. a subset of the full activity range (grey zone, where dead time is less than 1%) as per Method A. Data is shown for each detector separately, and averaged

**Fig. 6:**
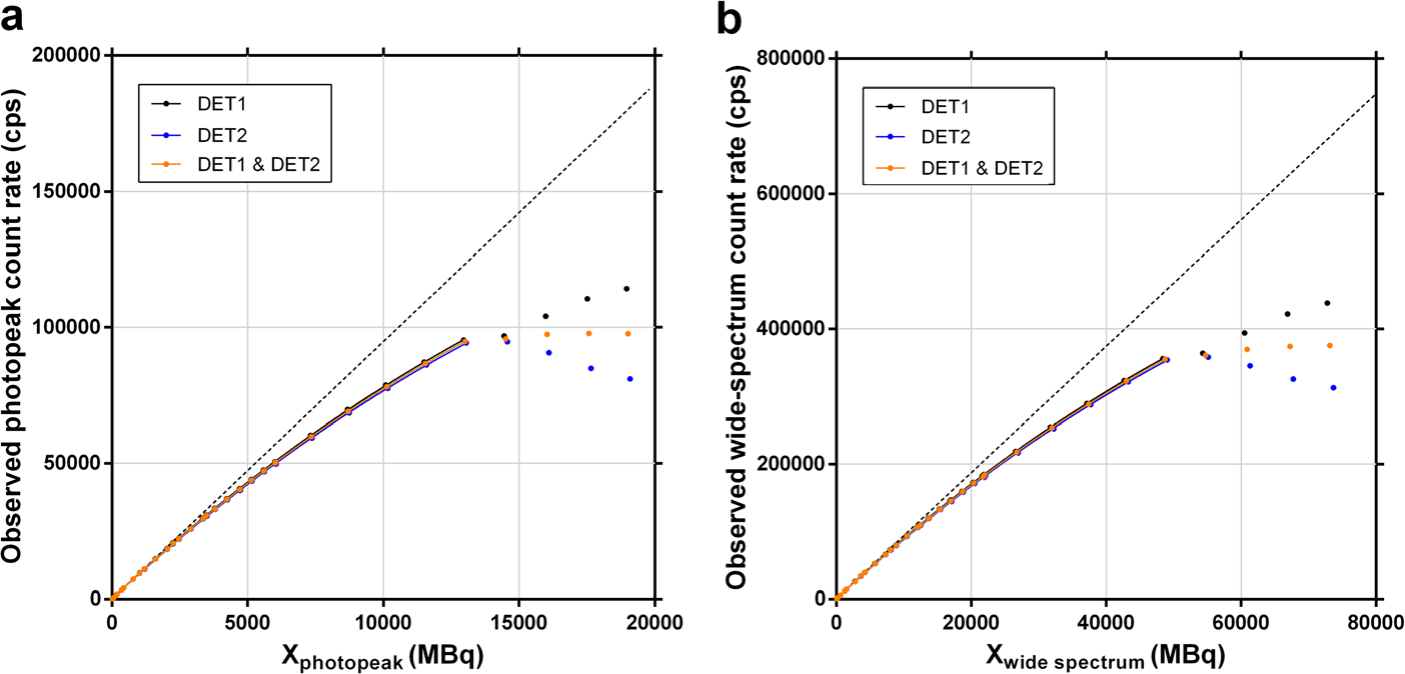
Observed acquisition photopeak (**a**, 187–229 k) and wide-spectrum (**b**, 18–680 keV) count rates vs. *X*_*W*_ . The term *X*_*W*_ is the product of activity and observed acquisition count rate in a given window (photopeak or wide spectrum, respectively), divided by the observed primary photon count rate. Solid lines represent the non-linear curve fit forced through origin of Eq. 3 to the data in the usable system range, allowing to resolve the camera calibration factor and the dead-time constant as per Method B (full-range *CF*). Data is shown for each detector separately, and averaged. Dotted lines represent the average calibration factor for both detectors (**a**, 9.40 cps/MBq; **b**, 9.36 cps/MBq)

- Method A (low-activity *CF*): *CF* was estimated by a linear fit forced through origin of *R*_*Po*_ vs. activity, at low activity (< 500 MBq; Fig. 5), yielding 9.38 cps/MBq (both detectors averaged). With *CF* fixed, *R*_*Wo*_ was plotted against the term *X*_*W*_ for both the photopeak and the *6W* wide-spectrum count rate, yielding τ of 2.1 μs and 0.56 μs, respectively (Table 3; graphs are not shown, as they are virtually identical to Fig. 6).
- Method B (full-range *CF*): *R*_*Wo*_ was plotted against the term *X*_*W*_ for both the photopeak and the *6W* wide-spectrum count rates (Fig. 6). Fitting Eq. 3 to the data allowed to resolve *CF* and τ (Table 3). Method B, applied to planar images wide-spectrum count rate, yielded *CF* and τ values within 0.2% and 1.2%, respectively, of those derived from Method A, without the need to arbitrarily determine a DT-free subset of data.

**Table 3:** Calibration factor and dead-time constant (± SE) derived from planar images

No. of acquisitions Activity range (MBq) Photopeak count rate range (cps) Wide-spectrum count rate range (cps)		Detector	Calibration factor (cps/MBq)			Dead-time constant (μs)			
			Method A	Method B Photopeak	Method B Wide-spectrum	Method A Photopeak	Method B Photopeak	Method A Wide-spectrum	Method B Wide-spectrum
Low activity range	Full activity range								
7	30	1	9.42 ± 0.02	9.49 ± 0.01	9.44 ± 0.01	2.00 ± 0.01	2.06 ± 0.01	0.541 ± 0.001	0.547 ± 0.002
19–400	19–10,809	2	9.35 ± 0.03	9.32 ± 0.01	9.27 ± 0.02	2.13 ± 0.01	2.10 ± 0.01	0.573 ± 0.002	0.553 ± 0.004
208–4049	208–94,800	1 and 2	9.38 ± 0.03	9.40 ± 0.01	9.36 ± 0.01	2.07 ± 0.01	2.08 ± 0.01	0.557 ± 0.002	0.550 ± 0.003
850–14,400	850–355,034								

Method A refers to obtaining the calibration factor at low activity, i.e. a subset of the full activity range (Eq. 1), then obtaining the dead-time constant over the full activity range by fitting the paralysable model to the data (Eq. 3). Method B refers deriving both the calibration factor and the dead-time constant through a single curve fit with data from the full activity range (Eq. 3). The acquisition count rate from either the photopeak or the wide spectrum (*6W*, 18*–*680 keV, Table 2) was considered for dead-time constant determination

**Fig. 7:**
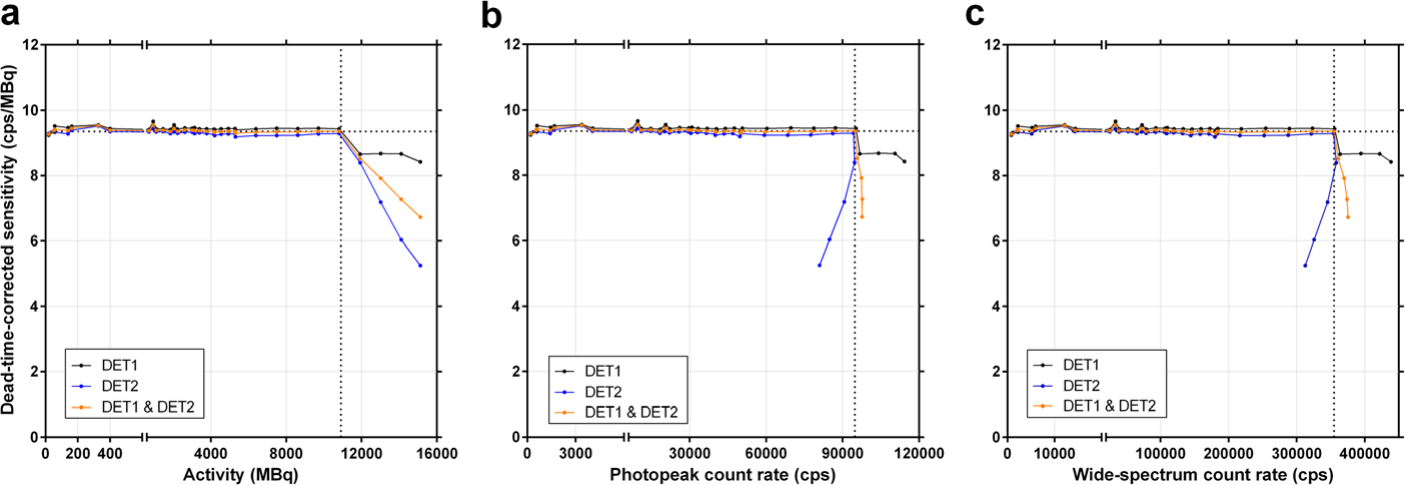
Dead-time-corrected planar sensitivity (observed primary photons count rate per activity) in function of activity (**a**), photopeak count rate (**b**, 187–229 keV) and wide-spectrum count rate (**c**, 18–680 keV). The horizontal dotted lines indicate the averaged calibration factor for both detectors (9.4 cps/MBq). The vertical dotted lines indicate the last point within the usable operating ranges of the system. Data is shown for each detector separately, and averaged

When plotting the DT-corrected planar sensitivity, using τ determined with Method B, the resulting curve behaviour confirms that the camera *CF* does not depend on the scanned activity and remains constant within the usable ranges of activity, photopeak and wide-spectrum count rates (Fig. 7). With *CF* and τ obtained with Method B, the average accuracy for recovering non-attenuated activity from DT-corrected planar images was 0.25 ± 0.62%.

### Tomographic-derived calibration factor and dead-time constant

**Fig. 8:**
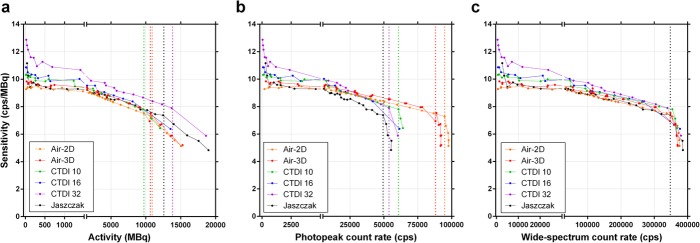
SPECT sensitivity (observed primary photons count rate per activity) as a function of activity (**a**), photopeak count rate (**b**, 187–226 keV) and wide-spectrum count rate (**c**, 18–680 keV). The vertical coloured dotted lines indicate, for each phantom, the maximum quantifiable activity and usable photopeak count rate, which are both geometry-dependant (**a** and **b**, respectively; those of the CTDI-16 phantom could not be precisely determined due to missing points at high activity). The vertical black dotted line corresponds to the unique, geometry-independent maximum wide-spectrum count rate of the SPECT system (**c**; ~350 kcps). Planar data (Air-2D, averaged for both detectors) is shown for comparison

Using *R*_*Po*_ of the entire reconstructed SPECT volume, the sensitivity was plotted against the activity, the acquisition photopeak and wide-spectrum count rates (Fig. 8). Unlike in planar mode, there was an upward tailing of tomographic sensitivity at low activity, which was more prominent for CTDI-32, the phantom having the largest volume of non-radioactive attenuating medium. Also, the maximum usable acquisition photopeak count rate (i.e. the point where sensitivity abruptly drops; Fig. 8b) appears clearly dependant on phantom geometry (volume of attenuating and scattering medium), while the maximum usable wide-spectrum count rate converges at approximately 350 kcps, regardless of geometry, and as it did in planar mode (Fig. 8c). This points towards the wide-spectrum count rate being a more appropriate, geometry-independent determinant of DT, as opposed to using the photopeak count rate for this purpose.

**Fig. 9:**
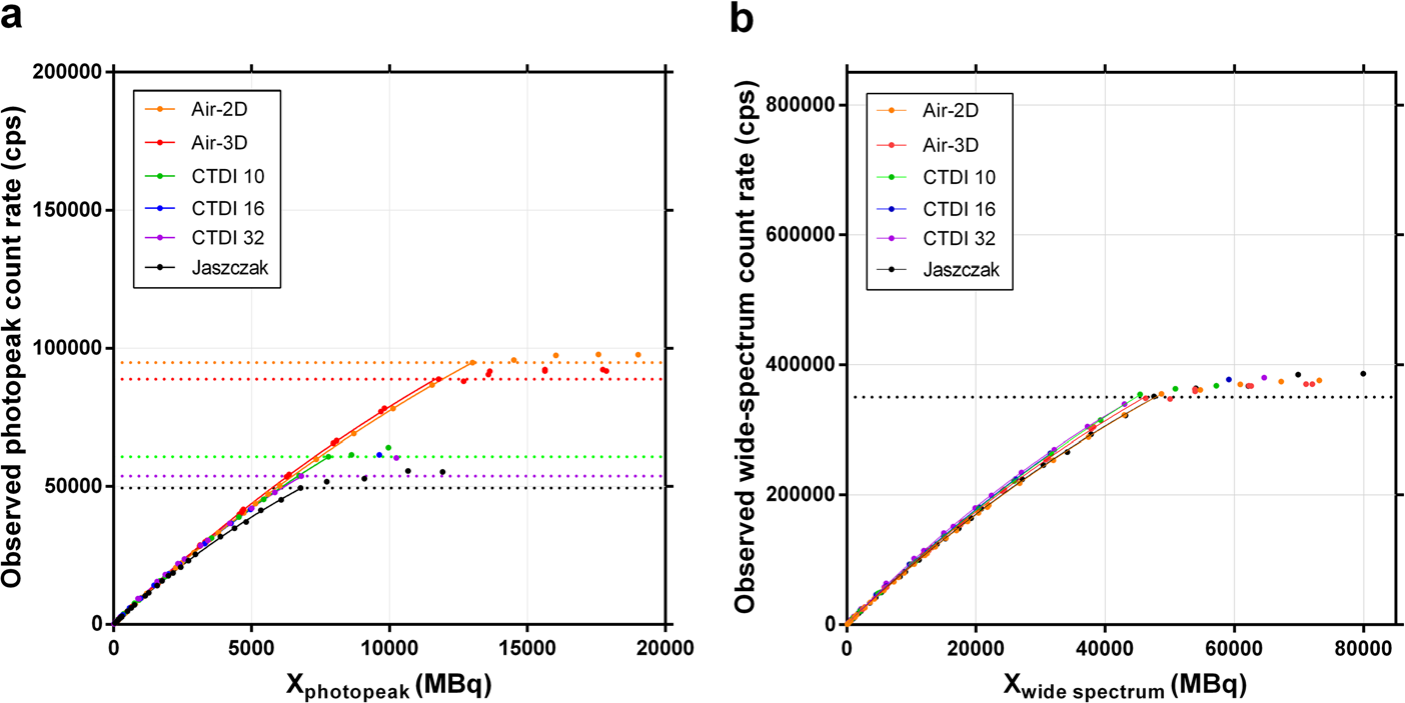
Observed photopeak (**a**, 187–226 keV) and wide-spectrum (**b**, 18–680 keV) count rates vs. *X*_*W*_. The term *X*_*W*_ is the product of activity and observed acquisition count rate (photopeak or wide-spectrum), divided by the observed primary photon count rate (Eq. 3). Coloured horizontal dotted lines indicate the maximum usable observed photopeak count rates, which is geometry-dependant (**a**; that of the CTDI-16 phantom could not be precisely determined due to missing points at high activity). The black horizontal dotted line corresponds to the unique, geometry-independent maximum wide-spectrum count rate (**b**). Planar data (Air-2D, averaged for both detectors) is shown for comparison

**Fig. 10:**
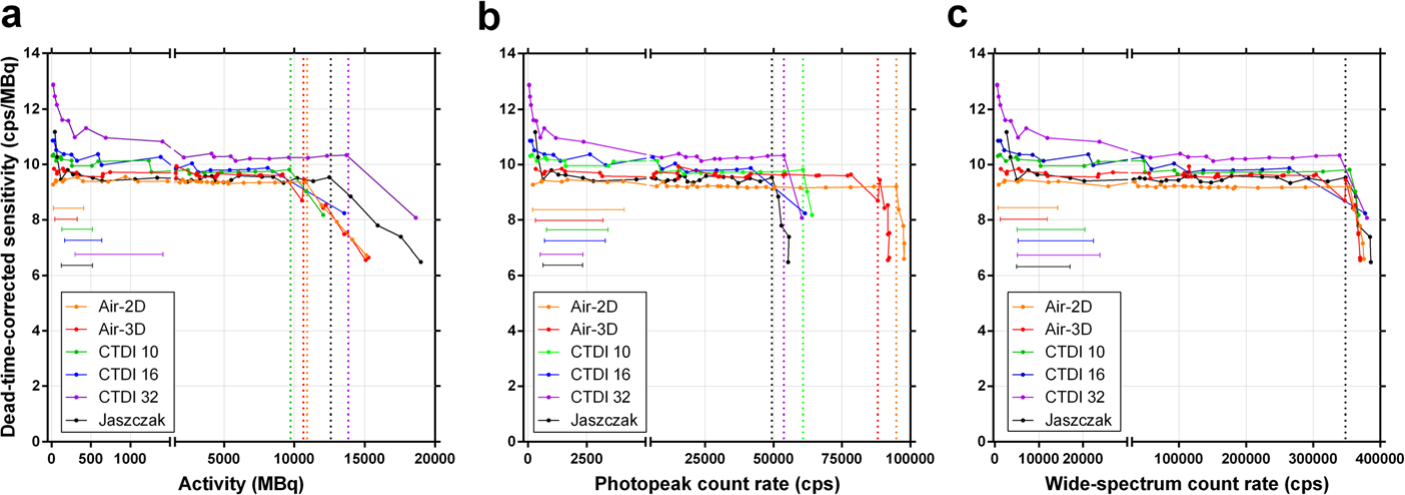
Dead-time-corrected SPECT sensitivity (observed primary photons count rate per activity) as a function of activity (**a**), photopeak count rate (**b**, 187–226 keV) and wide-spectrum count rate (**c**, 18–680 keV). The vertical coloured dotted line indicates, for each phantom, the maximum quantifiable activity and usable photopeak count rate, which are both geometry-dependant (**a** and **b**, respectively; those of the CTDI-16 phantom could not be precisely determined due to missing points at high activity). The vertical black dotted line corresponds to the unique, geometry-independent maximum wide-spectrum count rate of the SPECT system (**c**; ~350 kcps). The capped horizontal lines show, for each phantom, the low-count rate acquisition data points that could be used for calibration factor determination with Method A (low-activity *CF*; Table 4), i.e. data points with less than 1% dead time but without excessive sensitivity overestimation (upward tailing) at very low count rate (Table 4). Planar data (Air-2D, averaged for both detectors) is shown for comparison

The SPECT data was processed using Methods A (low-activity *CF*) and B (full-range *CF*) for both the photopeak and the wide-spectrum (*6W*) acquisition count rates (Table 4). The DT curves are illustrated (Fig. 9, Method B), showing once again a clear geometry dependence of the photopeak *R*_*Wo*_ vs. *X*_*W*_ relationship, and thus τ. Conversely, there is a geometry independence of the wide spectrum *R*_*Wo*_ vs. *X*_*W*_, and τ. When DT correction is applied to the reconstructed SPECT, as expected, the sensitivity response curves flatten (Fig. 10).

**Table 4:** Calibration factor and dead-time constant (± SE) derived from SPECT data

Phantom	Low activity	Full activity range	Calibration factor (cps/MBq)			Dead-time constant (μs)			
	No. of acquisitions Activity range (MBq) Photopeak count rate range (cps) Wide-spectrum count rate range (cps)		Method A	Method B Photopeak	Method B Wide-spectrum	Method A Photopeak	Method B Photopeak	Method A Wide-spectrum	Method B Wide-spectrum
Air-3D	7 32-330 327-3154 1342-11,975	27 32-10,704 327-88,762 1342-347,060	9.62 ± 0.04	9.70 ± 0.03	9.67 ± 0.03	2.07 ± 0.01	2.15 ± 0.03	0.531 ± 0.003	0.544 ± 0.008
CTDI-10	4 126-510 800-3423 5229-20,267	19 13-9646 93-60,750 665-354,256	9.91 ± 0.06	9.72 ± 0.03	9.70 ± 0.03	3.18 ± 0.02	2.93 ± 0.04	0.546 ± 0.005	0.499 ± 0.008
CTDI-16	5 157-634 693-3302 5523-22,333	16 16-80,94 83-41,651 694-264,231	9.98 ± 0.09	9.84 ± 0.05	9.85 ± 0.06	3.77 ± 0.06	3.28 ± 0.12	0.603 ± 0.010	0.528 ± 0.024
CTDI-32	4 293-1404 522-2364 5226-23,734	20 21-13,708 50-53,746 513-339,364	10.66 ± 0.09	10.19 ± 0.05	10.23 ± 0.04	4.47 ± 0.08	3.71 ± 0.07	0.705 ± 0.011	0.594 ± 0.012
Jaszczak	5 120-526 624-2335 4972-17,052	26 44-12,480 307-49,376 2650-348,334	9.50 ± 0.05	9.40 ± 0.05	9.46 ± 0.04	4.07 ± 0.04	4.04 ± 0.09	0.572 ± 0.005	0.565 ± 0.010

Method A refers to obtaining the calibration factor at low activity, i.e. a subset of the full activity range (Eq. 1), then obtaining the dead-time constant over the full activity range by fitting the data to the paralysable model (Eq. 3). Method B refers deriving both the calibration factor and the dead-time constant through a single curve fit using data from the full activity range (Eq. 3). The acquisition count rate from either the photopeak or the wide spectrum (*6W*, 18*–*680 keV, Table 2) was considered for dead-time constant determination

When applying *CF* and τ computed for each tomographic phantom individually, or these parameters derived from planar calibration (using Method B—full-range *CF*), the average accuracy for quantifying the total activity in the entire field of view (i.e. unsegmented) for all tomographic acquisitions was 0.47 ± 2.23% and − 0.02 ± 2.60%, respectively. For the Jaszczak phantom, which is considered the most clinically relevant geometry (activity dispersed in a large volume of attenuating medium), and using the planar-derived factors, the quantitative accuracy was 0.71 ± 1.18%.

### Segmentation

**Fig. 11:**
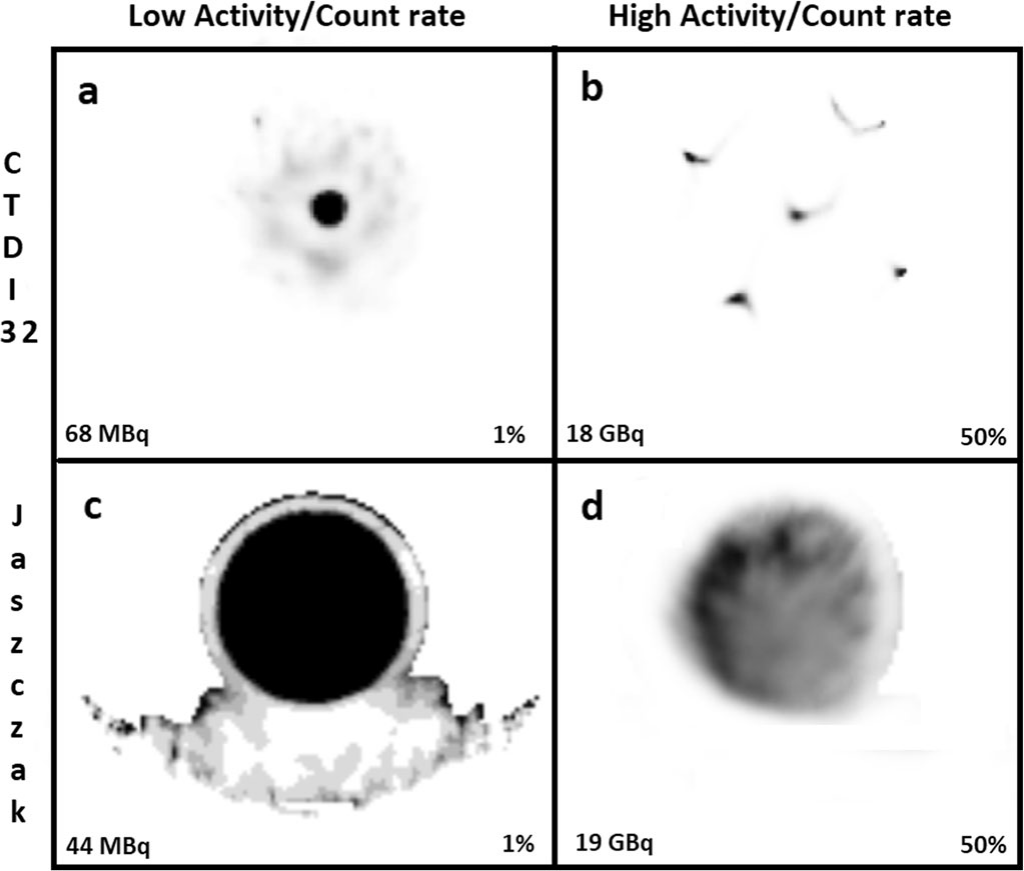
Artefacts on SPECT images of CTDI-32 (**a** and **b**) and Jaszczak (**c** and **d**) phantoms. Background activity is seen in areas of non-radioactive dense medium and the phenomenon is relatively more pronounced at very low activity (**a** and **c**). Streak (**b**) and non-uniformity (**d**) artefacts were see at very high count rates that were above the usable rage of the system. The images are normalized to the percentage of maximum voxel value indicated in each panel

**Fig. 12:**
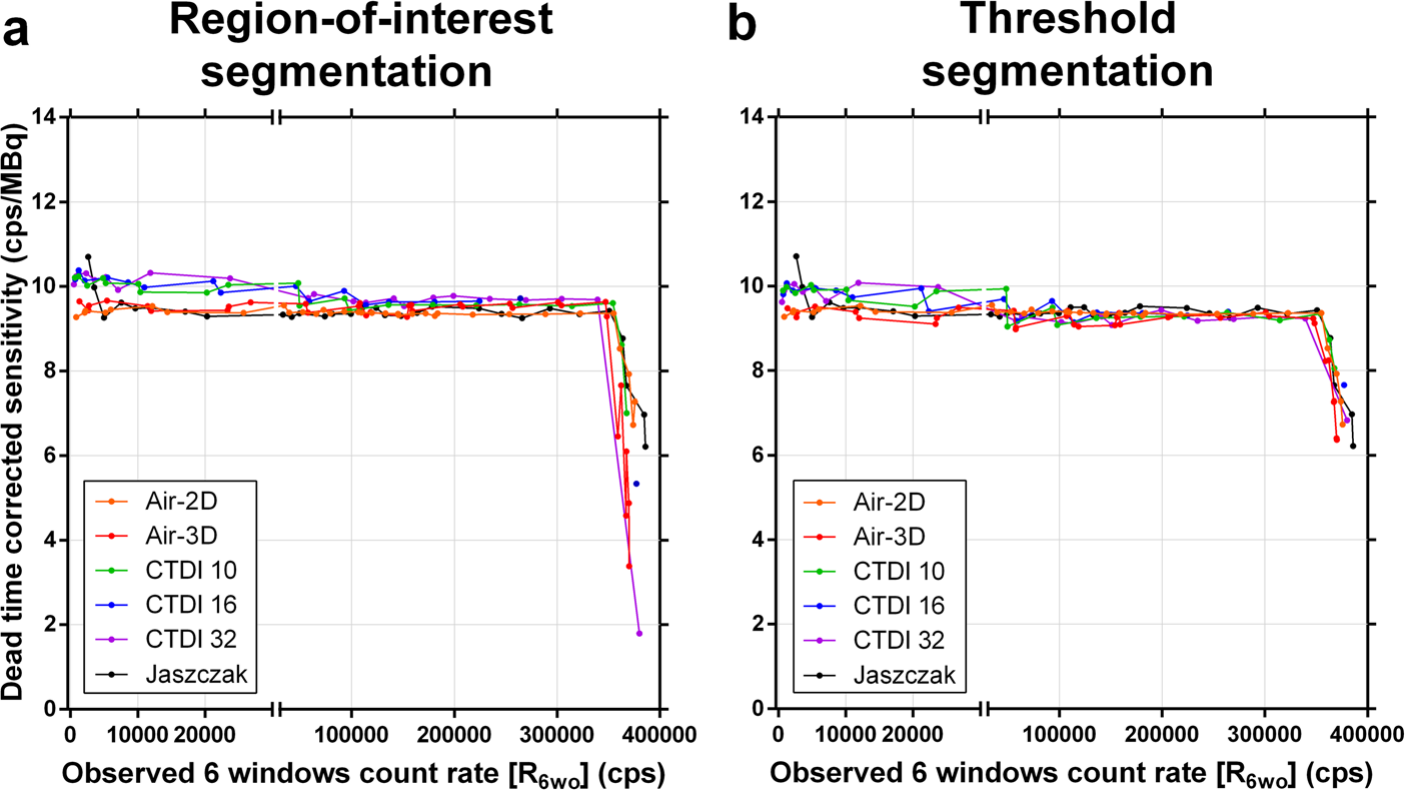
Dead-time-corrected SPECT sensitivity (observed primary photons count rate per activity) as a function of observed wide-spectrum count rate when activity segmentation was applied to the reconstructed SPECT images by drawing circular regions of interest around source regions (**a**), or by thresholding at one percent of maximum voxel (**b**). Planar data (Air-2D, averaged for both detectors) is shown for comparison

Characteristic artefacts could be observed in the reconstructed SPECT images (Fig. 11). In particular, in SPECT, excessive low-level background activity was seen in areas of non-radioactive dense medium, e.g. CTDI phantoms, Jaszczak phantom wall, and camera bed (Fig. 11a, c). This phenomenon is likely due to suboptimal scatter correction and was more pronounced with increased CTDI phantom size and at low activities or count rates [20]. This resulted in overestimated sensitivity when compiling the primary photons count rate from the entire SPECT field of view (Table 4, Figs. 8 and 10). To compensate for this phenomenon by eliminating the spurious background counts, segmentation of activity was applied to SPECT images using two techniques (ROI and threshold techniques) and data was re-analysed. *CF* and τ were determined for each segmentation technique and phantom (Table 5), and DT-corrected sensitivity was plotted against *R*_*Wo*_ (Fig. 12). For both segmentation techniques, sensitivity overestimation, including the upward tailing at very low activity, was greatly diminished. Furthermore, *CF* and τ were much less geometry-dependant, in particular for the CTDI phantoms, and tended to converge towards the planar *CF* and τ. In particular, the *CF* and τ of the segmented Jaszczak phantom were within 1% and 3%, respectively, of the planar data.

**Table 5:** Camera calibration factor and dead-time constant (± SE) derived from segmented SPECT images

Phantom	18–680 keV wide spectrum & TEW			
	Region-of-interest segmentation		Threshold segmentation	
	Calibration factor (cps/MBq)	Dead-time constant (μs)	Calibration factor (cps/MBq)	Dead-time constant (μs)
Air-3D	9.51 ± 0.06	0.570 ± 0.014	9.25 ± 0.05	0.577 ± 0.012
CTDI-10	9.58 ± 0.04	0.535 ± 0.010	9.30 ± 0.07	0.578 ± 0.016
CTDI-16	9.68 ± 0.06	0.522 ± 0.023	9.36 ± 0.08	0.512 ± 0.032
CTDI-32	9.69 ± 0.04	0.538 ± 0.009	9.25 ± 0.05	0.516 ± 0.016
Jaszczak	9.39 ± 0.04	0.568 ± 0.011	9.38 ± 0.05	0.565 ± 0.013

Method B (full-range *CF*), with wide-spectrum (18–680 keV) acquisition count rate, was used to derive the calibration factor and the dead-time constant. TEW: Triple-energy window scatter correction

When applying *CF* and τ derived from planar calibration (using Method B—full-range *CF*), the average accuracy for quantifying the total activity in the ROI-segmented or Threshold-segmented tomographic images was 0.09 ± 2.53% and 0.04 ± 2.31%, respectively. For the Jaszczak phantom only, it was 0.24 ± 1.06% and 0.02 ± 1.10%, respectively.

### Impact of the width of wide-spectrum window and scatter correction method

**Fig. 13:**
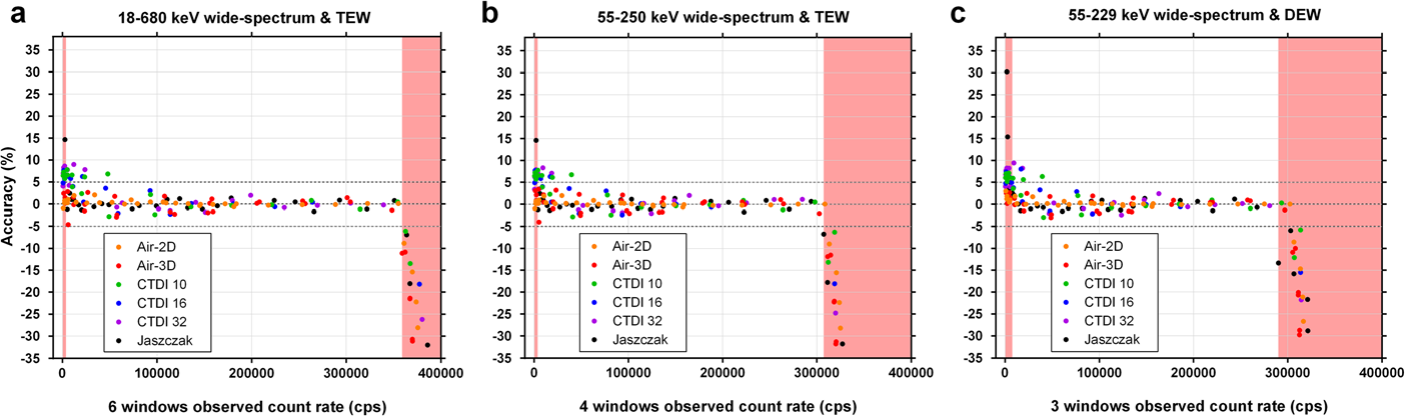
Accuracy of recovered activity (calculated with phantom-specific calibration factor and dead-time constant) from dead-time-corrected planar and SPECT images using 3 different energy window widths for wide-spectrum count rate monitoring: 18–680 keV (**a**) to 55–250 keV (**b**), and to 55–229 keV (**c**). TEW (**a** and **b**) or DEW (**c**) scatter correction was used. The red shaded regions indicate the count ranges where errors were above 10% occurred

**Fig. 14:**
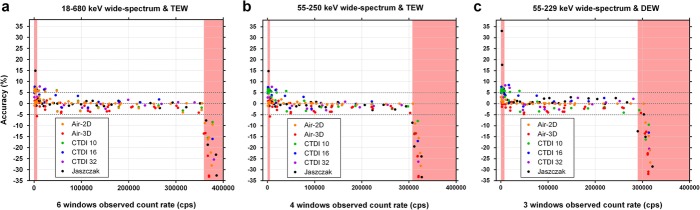
Accuracy of recovered activity (calculated with common calibration factor and dead-time constant derived from planar data) from dead-time-corrected planar and SPECT images using 3 different energy window widths for wide-spectrum count rate monitoring: 18–680 keV (**a**) to 55–250 keV (**b**), and to 55–229 keV (**c**). TEW (**a** and **b**) or DEW (**c**) scatter correction was used. The red shaded regions indicate the count ranges where errors above 10% occurred

The span of the wide-spectrum *R*_*Wo*_ monitoring window *W* was successively reduced from 18–680 keV (*6W*, Table 2) to 55–250 keV (*4W*, Table 6), and to 55–229 keV (*3W*, Table 6) by limiting the number of summed acquisition energy windows. TEW (*6W* and *4W*) or DEW (*3W*) scatter correction method was used to obtain *R*_*Po*_ for planar acquisitions (Air-2D) and segmented SPECT reconstructions (threshold-based segmentation; Method B—full-range *CF* and τ determination). Whether using phantom-specific *CF* and τ (Fig. 13) or common *CF* and τ derived from the planar data (Fig. 14), the quantification appears similarly accurate between the *6W*, *4W* and *3W* schemes. However, the data points are slightly more dispersed around the identity line with the *3W* scheme that also includes DEW using the planar *CF* and τ (Fig. 14c). The red-shaded zones represent the ranges where quantification is less accurate (>10% error), because of overestimation at low count rate, or underestimation above the maximum usable count rate (Fig. 13 and 14). Average accuracy (± SD) of pooled data points between these boundaries, computed using planar *CF* and τ (Fig. 14) was: 0.04 ± 2.31% for *6W*/TEW, − 0.13 ± 2.29% for *4W*/TEW, and 0.26 ± 2.51% for *3W*/DEW.

**Table 6:** Camera calibration factor and dead-time constant (± SE) derived from planar and threshold-segmented SPECT data with reduced wide-spectrum window width

Phantom	55–250 keV wide spectrum and TEW		55–229 keV wide spectrum and DEW	
	Calibration factor (cps/MBq)	Dead-time constant (μs)	Calibration factor (cps/MBq)	Dead-time constant (μs)
Air-2D^a^	9.36 ± 0.01	0.632 ± 0.003	9.54 ± 0.01	0.534 ± 0.003
Air-3D	9.19 ± 0.05	0.638 ± 0.014	9.39 ± 0.05	0.565 ± 0.018
CTDI-10	9.30 ± 0.07	0.686 ± 0.019	9.48 ± 0.07	0.555 ± 0.021
CTDI-16	9.35 ± 0.08	0.606 ± 0.038	9.54 ± 0.08	0.462 ± 0.041
CTDI-32	9.31 ± 0.06	0.658 ± 0.020	9.44 ± 0.07	0.476 ± 0.026
Jaszczak	9.38 ± 0.05	0.673 ± 0.015	9.70 ± 0.06	0.496 ± 0.029

*TEW*: triple-energy window scatter correction. *DEW*: dual-energy windows scatter correction. Method B (full-range *CF*), with wide-spectrum acquisition count rate, was used to derive the calibration factor and the dead-time constant. ^a^Planar images of Air-2D phantom were not segmented, and data from both detectors was averaged

## Discussion

QSPECT is the state-of-the-art method for ^177^Lu internal dosimetry, either through QSPECT-only protocols, or hybrid methods relying on multiple time-points planar imaging in conjunction with one QSPECT to scale the planar-derived time-activity curves [29, 30]. Whereas careful *CF* determination is obviously key to accurate quantification, the DT correction improves the accuracy [8]. Our data and our experience with personalized PRRT show that DT must be compensated for to maximize accuracy of dose estimates, not only when administering high personalized activities, but also lower activities (such as 7.4 GBq) in patients with a very high tumour retention in whom the majority of activity can be contained within the SPECT field of view [7]. In our practice, the DT correction factor exceeds 1.05 in 15% of patients at 24 hours after injection, and values as high as 1.30 have been observed (i.e. up to 23% underestimation if no DT correction applied; full data will be published separately). We have developed practical methods to calibrate a SPECT/CT system for ^177^Lu-QSPECT, including DT correction based on wide-spectrum acquisition count rate, which we further refined here [8]. Furthermore, QSPECT data can be very conveniently viewed and analysed after converting the reconstructed SPECT volume into PET-like dataset (i.e. *PT* DICOM modality) in which the *rescale slope* factor that integrates acquisition time, voxel volume, *CF*, and DT correction factor is added to the DICOM header, which then allows for displaying images representing count data in units of Bq/mL or standardized uptake values.

It has been previously shown that QSPECT *CF* can be determined from SPECT or planar images [9, 17, 20]. Our results confirm that a planar-based calibration consisting of serial planar imaging of sources in air over the entire operational range of the system also enables to resolve τ to generate a lookup table of DT correction factor for primary (reconstructed) counts vs. wide-spectrum acquisition count rate. For both *CF* and τ, the agreement is particularly excellent between planar- and Jaszczak-derived values. We previously obtained a larger *CF* of 10.8 MBq/cps for the same SPECT/CT system, which was obtained using serial SPECT/CT acquisitions of point-sources, surrounded by attenuating medium in the majority of acquisitions [8]. The latter result is consistent with that obtained here with the CDTI-32 phantom (10.2 cps/MBq) with no segmentation applied, resulting in a slight *CF* overestimation. Our comprehensive study with a variety of phantoms shows that *CF* and τ derived from tomographic or planar acquisitions tend to converge when segmentation is applied to tomographic data. Indeed, TEW or DEW scatter correction is less effective to get rid of the background counts in areas of dense, non-radioactive medium, leading to overestimated sensitivity when background noise is not excluded by mean of segmentation [17, 20, 26, 31, 32]. We have a preference for the threshold-based segmentation method because the SPECT-derived *CF* tended to be more consistent among the various phantom geometries, as well as with the planar *CF*. While planar calibration is more practical for camera calibration, we recommend performing at least one or a few SPECT acquisitions, ideally of an attenuating phantom containing DT-generating activities, to validate the planar-derived parameters.

Our prior results, and more convincingly the data presented here, clearly show that a wide-spectrum count rate monitoring approach must be adopted to accurately correct for DT using a single, geometry-independent τ [8]. Indeed, photons of any energy recordable by the system generate DT and, for a given activity, the shape of the energy spectrum histogram is highly impacted by the volume of attenuating matter. While sources in air are not representative of a clinical situation, the CTDI and Jaszczak phantoms are informative in that regard. Patient geometry can vary from the air-filled thorax of a cachexic patient to the abdomen of an overweight patient. Furthermore, scatter from high activity outside the field of view (e.g. bladder or abdominal tumours while imaging the thorax) can cause DT that cannot be accurately estimated if monitoring only the photopeak or primary count rate. Wide-spectrum count rate is ideally monitored over the entire recordable range of energy (e.g. our *6W* range) or a range collecting the vast majority of counts (e.g. our *4W* range), for a given radionuclide and SPECT/CT system. It is important to emphasize that the τ obtained by our methods is not meant to estimate the DT affecting the wide-spectrum acquisition count rate itself (in which case we would have obtained 0.285 ± 0.004 μs here, similar to 0.19 ± 0.18 or 0.40 ± 0.25 μs obtained previously with the same system [21]), but only the DT affecting the primary count rate (i.e. those from the scatter-corrected planar or reconstructed SPECT images) that is also affected by pulse pile-up which amplifies the primary count losses relative to the wide spectrum. Besides, we found that TEW seems marginally more accurate than DEW (0.04% and − 0.13% for TEW, vs. 0.26% for DEW) as a scatter correction method, likely due to better subtraction of piled-up counts monitored by the upper scatter window.

The motivation to devise Method B (full-range *CF*) for *CF* and τ determination was to use the full range of clinically relevant ^177^Lu activities to calibrate the system with serial SPECT acquisitions [8]. Determining *CF* separately from τ, i.e. at low activity for which DT is assumed to be negligible, as per Method A (low-activity *CF*), may result in overestimation of *CF* because the background noise is ineffectively eliminated by scatter correction of tomographic data in low-count conditions. This is not an issue with planar calibration because TEW-correction of photopeak counts (i.e. primary counts) at the field of view level is effectively eliminating this background noise at low activity. But, even then, the activity level below which DT can be assumed to be negligible (as it is never null), needs to be determined arbitrarily. Determining both *CF* and τ in a single regression, as per Method B (full-range *CF*), is a practical way to circumvent this limitation.

SPECT quantification at very low count rate remains of limited accuracy, which is in agreement with Robinson et al. [31]. At high counting rate, the pile-up effect contributes to primary photon count losses in addition to the processing DT of the system. Our data clearly shows that the paralysable model based on wide-spectrum acquisition count rate is nevertheless able to accurately estimate lost primary photon counts regardless of the cause, up to the maximum usable wide-spectrum count rate (~ 350 kcps) of the system. As observed by others, the actual maximum count rate was lower than the theoretical one (1/*e* · 0.550 μs = 669 kcps for our system), which emphasizes the need to experimentally measure it to fully characterize the system [16]. Beyond that point quantification is not possible and, with our SPECT/CT system, the image is distorted because of the divergent detector behaviour (Fig. 5, 7, 8 and 12) [16, 24].

With its low abundance of medium-energy gamma emissions, ^177^Lu is an ideal therapeutic beta-emitting radionuclide for quantitative imaging, as compared to other common beta radionuclides such ^90^Y (bremsstrahlung challenging to quantify) or ^131^I (very high abundance of high-energy gammas). Practical calibration methods for existing SPECT/CT systems, along with simplification of dosimetry protocols, could accelerate clinical research and enable widespread practice of personalized ^177^Lu radionuclide therapy, which is likely to further improve the clinical benefits.

## Conclusion

We have shown that a comprehensive, accurate and practical calibration of a SPECT/CT system for ^177^Lu-QSPECT can be accomplished with serial planar acquisitions of sources in air. The latter must have a sufficient total activity to exceed the full operational counting range of the system in order to determine a reliable τ based on wide-spectrum count rate, as DT correction is essential for accurate quantification in a post-therapy setting, as well as to measure the maximum counting rate of the system. QSPECT enables fully exploiting the theranostic properties of ^177^Lu, which constitute a significant advantage over other therapeutic radionuclides.

## Data Availability

Please contact the corresponding author for the data used in this manuscript.

## Abbreviations

*CF*: Calibration factor
CTDI: Computed tomography dose index
DT: Dead time
DEW: Dual-energy window
PRRT: Peptide receptor radionuclide therapy
QSPECT: Quantitative single-photon emission computed tomography
ROI: Region of interest
TEW: Triple-energy window
